# Morphometrics of eight Chinese cavefish species

**DOI:** 10.1038/s41597-019-0257-5

**Published:** 2019-10-25

**Authors:** Enrico Lunghi, Yang Zhao, Xueying Sun, Yahui Zhao

**Affiliations:** 1 Key Laboratory of the Zoological Systematics and Evolution, Institute of Zoology, Chinese Academy of Sciences, Beijing, China; 2 Museo di Storia Naturale dell’Università degli Studi di Firenze, Sezione di Zoologia “La Specola”, Firenze, Italy; 3 College of Life Sciences, Hebei University, Baoding, China; 4 China University of Geosciences, Beijing, China

**Keywords:** Ichthyology, Biodiversity

## Abstract

Chinese cavefishes are a bizarre and interesting vertebrate taxa, but one with relatively little research. China holds the highest global cavefish diversity, accounting for about one-third of known species. *Sinocyclocheilus* is the largest genus of cavefishes in the world and is endemic to the south of China. The distribution of *Sinocyclocheilus* species is very narrow, and sometimes they inhabit just a single cave; this feature increases the vulnerability to extinction. With this study we provide the first comprehensive dataset related to the morphometrics of eight *Sinocyclocheilus* species. In addition to enhancing our knowledge on these poorly known species we aim to provide a dataset useful for future comparative analyses aiming to better understand the adaptive ability of cavefishes.

## Abstract (summary)

**Table:** 

Measurement(s)	morphometry
Technology Type(s)	digital camera • computational modeling technique
Factor Type(s)	species • Museum collection
Sample Characteristic - Organism	Sinocyclocheilus
Sample Characteristic - Environment	groundwater
Sample Characteristic - Location	China

Machine-accessible metadata file describing the reported data: 10.6084/m9.figshare.9963206

## Background & Summary

Cavefishes are one of the least studied vertebrate taxa globally^1,2^. These fish are restricted to groundwater environments (from here the name stygo-fauna)^3,4^, habitats which are generally difficult to explore, even for specialists^5,6^. Cavefishes show a range of adaptations to subterranean environments^7,8^, namely habitats characterized by particular features such as the general lack of light and limited availability of food resources^9,10^. Such adaptations may involve changes in fish behaviour and physiology, but also the development (or regression) of various organs and body shapes^2,11,12^. These adaptations are most evident in obligate cave species (stygobites) as they are normally not able to exit caves and thus, high adaptation degree to subterranean environments is beneficial for their fitness^13^. Contrary to this facultative cave-dwellers (stygophiles) show many fewer specialist adaptations to subterranean environments as they show a mixed lifestyle, alternating between hypogean and epigean phases^14,15^.

China hosts over 150 cavefish species, accounting for about one-third of known species worldwide^16^. South China Karst hosts the majority of Chinese cavefishes, and most of them are endemic to small areas or even to single caves, condition increasing their conservation concerns^16–18^. Chinese cavefishes mainly belong to the order Cypriniformes; only one species is ascribed to the order Siluriformes^8^. The genus *Sinocyclocheilus*, with more than 70 known species, is the worldwide largest known group of cavefishes and is endemic to South China^16,19^. Its diversity occurs in a relatively narrow area, suggesting high adaptability of these fishes to the subterranean environment^16,20^; indeed, more than half of *Sinocyclocheilus* species are stygobites^8^. Four different clades are recognized within *Sinocyclocheilus* fishes: “jii”, “cyphotergous,”, “tingi” and “angularis”^8,21^. In all four clades some fishes develop the humpback, a peculiar morphological shape thought to serve as energy storage in environments where food supply is not constant^8^. In the “angularis” clade a further bizarre onward projection (the so called “horn”) is also present, but its function is still unknown^16,22^.

*Sinocyclocheilus* fishes are an important component of both Chinese and worldwide biodiversity^16,19,20^; yet, almost no effort is dedicated to their protection^1,18^. Cave species are often very susceptible to environmental changes^23–25^ and their narrow distribution increases their vulnerability to extinction risk^26–28^. However a lack of clear information on the distribution or ecology of this taxa impedes effective conservation management and prioritisation^1,29,30^. Although the genetic and genomic of Chinese cavefishes were the topic of several scientific papers (Refs^21,31^ among them), no other studies on their ecology, behavior or life history exist.

In the present work, we report the most comprehensive dataset on the morphology of eight Chinese cavefishes belonging to the genus *Sinocyclocheilus* (*S*. *brevibarbatus*, *S*. *brevis*, *S*. *huanjiangensis*, *S*. *jii*, *S*. *lateristritus*, *S*. *mashanensis*, *S*. *microphthalmus* and *S*. *qiubeiensis*). At present information on these species is virtually absent, and the limited available data lies only in Chinese literature^19^. Our goal is therefore to raise awareness of Chinese cavefishes, providing useful data to be employed in future comparative analyses with other cavefishes; in this way, divergences and similarities in adaptive abilities across different species worldwide could be assessed. Our study will contribute in improving species knowledge, an important step towards species protection^29^. To do that, we started with sharing the information related to the specimens present in the collection of the Institute of Zoology of the Chinese Academy of Sciences in Beijing (China), which holds the biggest collection of Chinese cavefishes. Specimens from different species and populations are present in this collection and they were often used in taxonomic and phylogenetic studies.

## Methods

### Experimental design

**Fig. 1:**
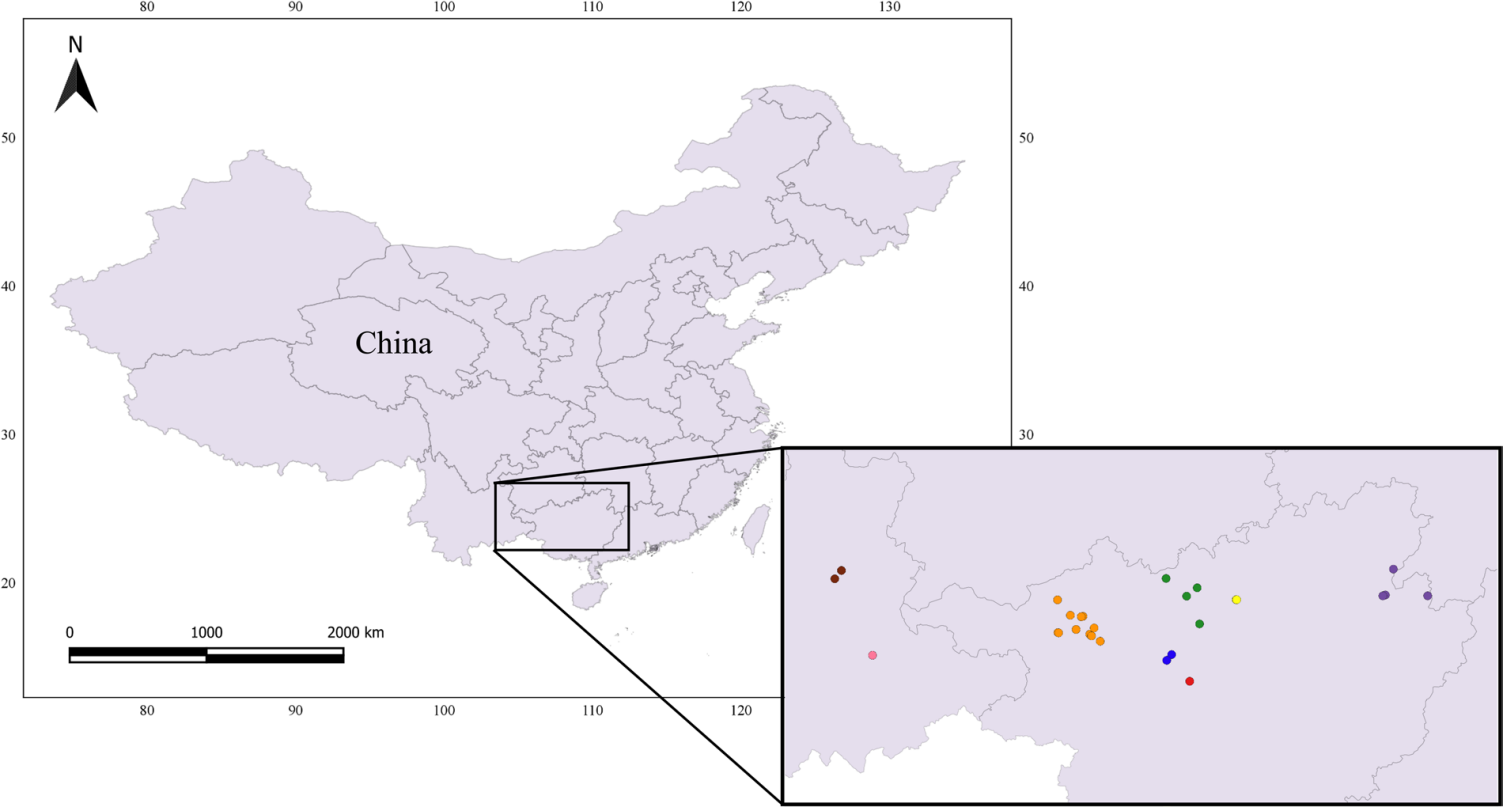
Map of the area from where the specimens were collected. Each symbol corresponds to a distinct population of the following species: *Sinocyclocheilus brevibarbatus* (blue), *S*. *brevis* (yellow), *S*. *huanjiangensis* (green), *S*. *jii* (violet), *S*. *lateristritus* (brown), *S*. *mashanensis* (red), *S*. *microphthalmus* (orange), *S*. *qiubeiensis* (pink). Maps were created with the program QGIS^37^ using data from http://www.ngcc.cn/ngcc//html/1/.

**Fig. 2:**
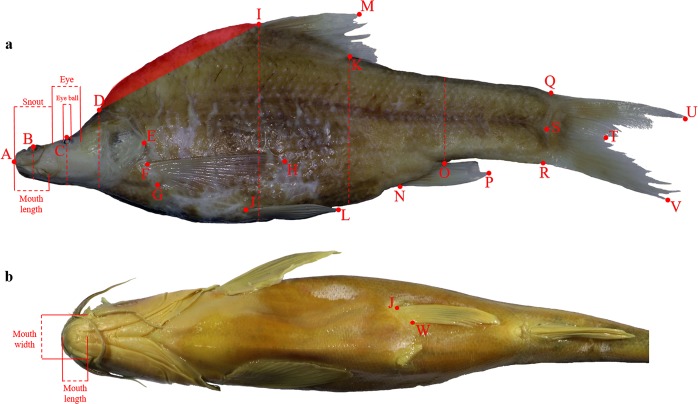
Reference adopted in fishes’ body measurements. In the figure two individuals of (**a**) *S. microphtalmus* and (**b**) *S*. *jii* showing all the landmarks and dashed lines used to record measurements on (**a**) the lateral and (**b**) the ventral side of the fish body (see the main text for a detailed description of the recorded measurements). Shaded area indicates the fish humpback.

We examined specimens belonging to 8 species of Chinese cavefishes from the collection of the Institute of Zoology of the Chinese Academy of Sciences in Beijing (China). The examined species inhabit groundwater environments in the Provinces of Guangxi (*N* species = 6) and Yunnan (*N* species = 2) (Fig. 1). We built up a large database including date and locality of fish collection, the description of their body organs and morphometrics. When precise coordinates were present, we provide a specific code (species initials + a number) to distinguish between different populations (Table 1). According to the standard methodology used to record fishes’ morphology^32^, we identified multiple landmarks from which measurements were taken (Fig. 2). These points correspond to the following body parts: A (snout tip); B (nostril); C (eye); D (top end of the head); E (farthest backward end of the head); F (beginning of the forward pectoral fin base); G (end of the forward pectoral fin base); H (farthest end of the forward pectoral fin lobe); I (beginning of dorsal fin base); J (beginning of the backward pectoral fin base); K (end of dorsal fin base); L (farthest end of the backward pectoral fin lobe); M (farthest end of the dorsal fin lobe); N (beginning of the anal fin base); O (end of the anal fin base); P (farthest end of the anal fin lobe); Q (top beginning of caudal fin); R (low beginning of caudal fin); S (middle point between Q and R); T (median end of the caudal fin lobe); U (farthest end of the top caudal fin lobe); V (farthest end of the low caudal fin lobe); W (end of the backward pectoral fin base). Alongside measurements involving the above listed points (see below), we also recorded data from additional parts of fishes’ body (identified with dashed lines, Fig. 2): Snout (distance between the mouth tip and the beginning of the eye); Eye (eye diameter); Eyeball (eyeball diameter); Mouth width (length between the two mouth angles); Mouth length (length of the lower jaw).

**Table 1:** Morphometrics of eight Chinese cavefishes. Detailed information related the morphometrics of the eight *Sinocyclocheilus* species^33^.

Column	Data description	Typology of data
1	Collection_ID	The specimen’s code in the ASIZB collection
2	Family	The specimen’s family
3	Genus	The specimen’s genus
4	Species	The specimen’s species name
5	Country	The country of specimen collection
6	Province	The province of specimen collection
7	County	The county of specimen collection
8–9	Latitude and Longitude	Downgraded coordinates of collection localities
10–11	Month and Year	The date of specimen collection
12	Population	Code of each cavefish population
13	Eye	Indicates if the eye is well Developed, Reduced or Absent
14	Mouth_position	Indicates the position where the mouth opens:, Terminal, Subterminal, Inferior, Superior
15	Caudal_fin_shape	Indicates the shape of the fish caudal fin: Rounded, Truncate, Emarginate, Forked, Lunate
16–44	Measurement typology	The recorded measurements of cavefishes. Lengths are recorded in mm, while the area in mm^2^

### Specimens sampling

**Fig. 3:**
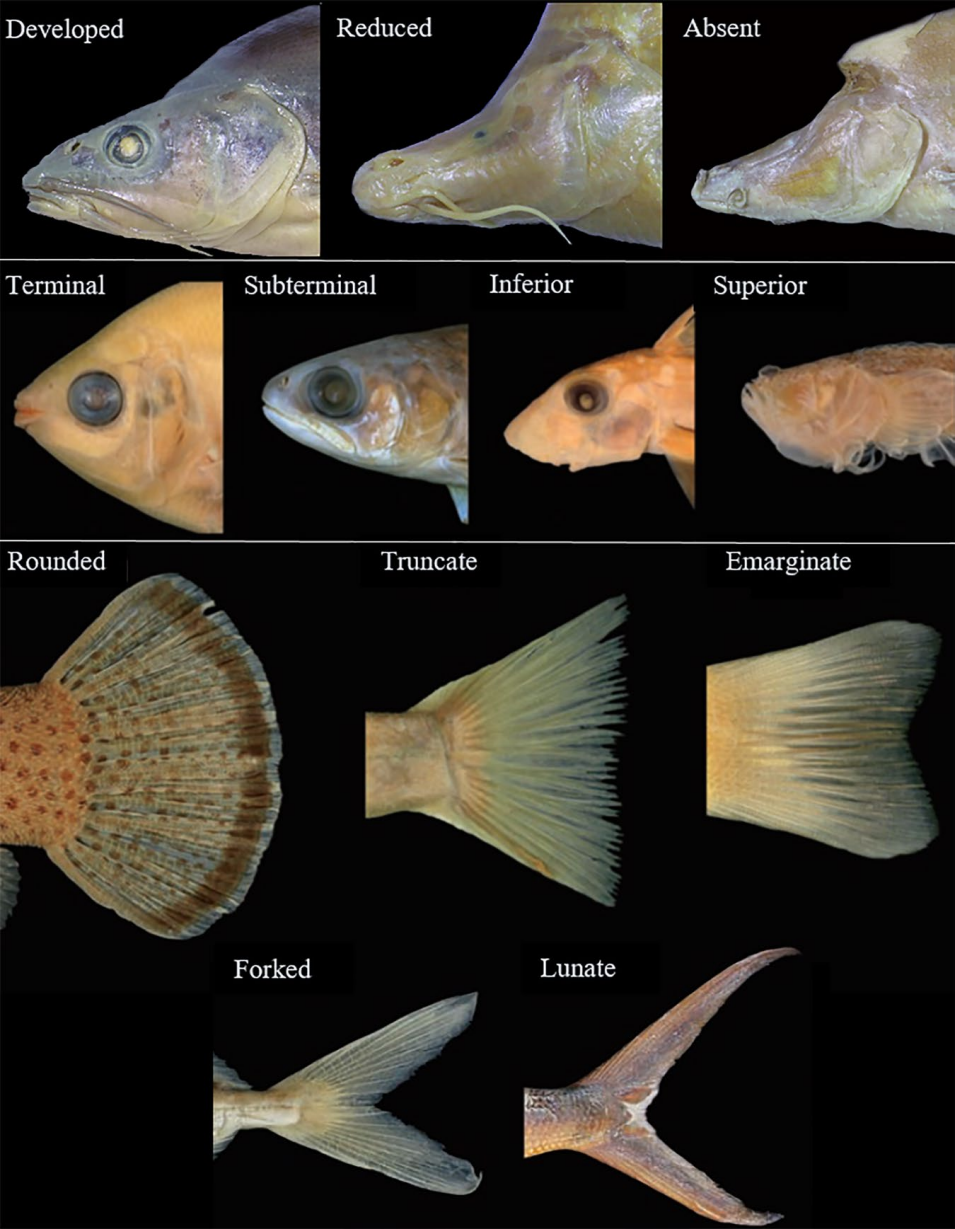
Reference showing the different shapes of the considered cavefish organs. Eye: Developed, Reduced, Absent. Mouth: Terminal, Subterminal, Inferior, Superior. Caudal fin: Rounded, Truncate, Emarginate, Forked, Lunate. Images for mouth and caudal fin are modified from^38^.

We first described the shape of three body organs: the eye, the mouth and the caudal fin. For the eye, we considered three different categories according to the eye ball’s development degree: “Developed” when is fully developed; “Reduced” when is small and poorly developed; “Absent” when the fish lack eyes (Fig. 3). We then described the mouth position according to where the opening occurs: “Terminal” if it opens at the tip of the fish head; “Subterminal” if it opens close to the tip head but downward; “Inferior” if it opens downward; “Superior” if it opens upward (Fig. 3). We finally described the caudal fin according to its shape. We used five different shape categories: “Rounded”, “Truncate”, “Emarginate”, “Forked”, “Lunate” (Fig. 3).

After the first descriptive part, we recorded measurements of the fishes’ body parts. Measurements were taken using a digital calliper and analysing pictures of specimens. Digital calliper was used to record measurements hardly visible form pictures; in the following table, morphometrics recorded using this methodology are indicated with the symbol “*”. Pictures were taken using a digital camera and placing fishes on a light background with a ruler as a scale. Files were then analysed with the software ImageJ. Once the scale was settled, the distance between two points (Fig. 2) was measured with a straight line; the same method was used to evaluate the length of dashed lines (Fig. 2).

The recorded measures were the following:

- Eye*;
- Eye_ball*;
- Snout*;
- Mouth width*;
- Mouth length*;

- AD: linear distance between the snout tip and the top end of the head;
- B_height: head height measured at the nostril;
- C_height: head height measured at the eye;
- D_height: head height measured at the upper end;
- DI: linear distance between the top end of the head and the beginning of the dorsal fin;
- AE: maximum head length, measured from the snout tip until the farthest backward end of the head;
- FG: length of the forward pectoral fin base;
- FH: maximum extension of the forward pectoral fin;
- IM: maximum extension of the dorsal fin;
- IK: length of the dorsal fin base;
- I_depth: body depth measured at the beginning of the dorsal fin base;
- JL: maximum extension of the backward pectoral fin;
- JW: length of the backward pectoral fin base*;
- K_depth: body depth measured at the end of the dorsal fin base;
- NO: length of the anal fin base;
- O_depth: body depth measured at the end of the anal fin base;
- NP: maximum extension of the anal fin;
- QR: caudal fin height at its base;
- QU: maximum extension of the top part of the caudal fin;
- ST: caudal fin mid length;
- RV: maximum extension of the lower part of the caudal fin;
- AS: standard length;
- AT: total length.

Besides the above mentioned fish standard lengths, we recorded the measurement of a specific body part characterizing Chinese cavefishes: the humpback area^8^. This peculiar structure develops on the fish back, between the head and the dorsal fin (Fig. 2), and it is used to store energy, a practical adaptation to food deprived environments^8,9^. The humpback area (DID) is located above the DI segment (shaded area in Fig. 2a) and was delimited connecting back D from I following the animal shape.

## Data Records

The dataset (Morphometrics of eight Chinese cavefishes^33^) consists of:

1. 451 specimens belonging to eight *Sinocyclocheilus* species of Chinese cavefishes (*S*. *brevibarbatus N* = 34, *S*. *brevis N* = 31, *S*. *huanjiangensis N* = 42, *S*. *jii N* = 140, *S*. *lateristritus N* = 44, *S*. *mashanensis N* = 16, *S*. *microphthalmus N* = 101, *S*. *qiubeiensis N* = 43).
2. Description of three organs: eye, mouth and caudal fin.
3. Measurements of 28 fish body parts (27 in four species because their eye diameter equals the eye ball diameter).
4. NA means no specific data existing. Preserved specimens were not always integer or in some cases, after their fixation in alcohol, their original shape was not well conserved. This was also used in the category “Eye” when eye diameter equals the diameter of the eye ball. Furthermore, NA was used in the “Population” column to indicate that precise coordinates were not present.

Detailed explanation of dataset Morphometrics of eight Chinese cavefishes^33^ is given in Table 1.

## Technical Validation

Studied specimens belong to the fish collection of National Zoological Museum, the Institute of Zoology, Chinese Academy of Sciences (ASIZB)^34^; with an appropriated request, the same fishes can be further studied. Blinded fish measurements were performed to further reduce any possible bias^35^. The whole dataset was double-checked for any possible error. Outliers were identified in two ways: before by visual check (i.e., plotting the data), and then using three times the standard deviation from the data mean (+/−) as cut-off. Successively, the relative measurement was taken again to check whether the outlier was due to measurement mistakes.

## Usage Notes

Dataset is provided in CSV format, ready to be used with statistic programs like R (http://www.R-project.org/) and PAST. Precise coordinates of collection points are not shown to increase species protection^36^. Data were collected with instruments allowing high precision (0.01 mm). Prior to any analyses, we suggest to log-transform the measures to improve linearity and reduce skewness.

## Data Availability

No code was used in this study.
